# Ultra-high b-value diffusion-weighted imaging features of the prostatic leiomyoma-case report

**DOI:** 10.1186/s12880-017-0234-4

**Published:** 2017-12-20

**Authors:** Yanguang Shen, Yan Zhong, Haiyi Wang, Lu Ma, Yingwei Wang, Jinjin Pan, Zhonghua Sun, Huiyi Ye

**Affiliations:** 1 Department of Radiology, Chinese PLA General Hospital, Fuxing Road NO.28, Box 100853, Beijing, China; 2 Department of Medical Radiation Sciences, Curtin University, Perth, 6102 Australia

**Keywords:** Prostate, Leiomyoma, Magnetic resonance imaging, Diffusion weighted imaging

## Abstract

**Background:**

Leiomyoma of the prostate is a rare benign tumor arising from smooth muscle fibers. Most cases are incidental findings observed during pathological examinations after resection of the prostate. To the best of our knowledge, only few studies have reported the conventional magnetic resonance imaging (MRI) findings of such tumors; however, no reports have described the ultra-high b-value diffusion-weighted imaging (DWI) and apparent diffusion coefficient (ADC) findings of prostatic leiomyomas.

**Case presentation:**

We report MR imaging characteristics and surgical pathologic findings of a case of prostatic leiomyoma treated by robot-assisted transperitoneal laparoscopic approach. Typical MR features showed a homogeneous lesion with slightly hypointense signal compared to the skeletal muscle on T2-weighted images, and isointense signal relative to the muscle on T1-weighted images with fat suppression, which collectively demonstrate apparent homogeneous enhancement with a non-enhanced envelope. A slightly hyperintense signal compared to the skeletal muscle was observed on ultra-high b-value DWI, and higher ADC values were observed as compared to the prostate cancer.

**Conclusions:**

Prostatic leiomyoma is a benign tumor. This case indicates that MRI features of prostatic leiomyoma are helpful for the differential diagnosis of prostate cancer.

## Background

Leiomyoma of the prostate is a rare benign tumor arising from smooth muscle fibers. Most cases are incidental findings observed during anatomopathological examinations after a resection of the prostate [1]. Prostatic hyperplasia often includes leiomyomatous nodules [2]. They are most often removed surgically. To the best of our knowledge, only six reports have described the conventional MRI findings of such tumors, and no studies have presented the ultra-high b-value diffusion-weighted imaging and ADC findings of prostatic leiomyomas [3, 4]. Here, we present MRI findings of a patient with benign prostate hypertrophy and prostate cancer who was incidentally diagnosed with a prostatic leiomyoma. Ultrasound guided systematic transperineal prostatic biopsy and subsequent histological examination of biopsy samples confirmed prostatic cancer in the prostatic left zone (Gleason score 3 + 3 = 6). The patient was operated successfully by a robot-assisted laparoscopic radical prostatectomy.

## Case presentation

A 76-year-old man was referred to our urology department with repeated symptoms of dysuria, frequent urination, urgency, and painful micturition for one month. His past medical history showed no hypertension, diabetes, or any history of surgery.

Laboratory data on admission showed that the total prostate specific antigen level was 7.76 ng/ml, free prostate specific antigen level was 0.618 ng/ml, and white blood cell count was 10,750/mm^3^; however, urinalysis and blood chemistry were all within normal range. Digital rectal examination revealed an enlarged prostate, a prominent median sulcus, a smooth rubbery consistency with no palpable nodule, and elastic enlarged prostate.

Ultrasound scan showed an enlarged prostate (38 mm in anteroposterior diameter, 43 mm in longitudinal diameter, and 51 mm in transverse diameter), prostatic hyperplasia, and a gland of 43 g.

Patient’s pelvis was imaged in 3.0-T MRI (Discovery 750, GE Healthcare, Milwaukee, WI, USA) using a surface phased array coil. Conventional MRI sequences included both axial fast spin-echo T2-weighted imaging (T2WI) and DWI. T2WI of the entire prostate and seminal vesicles was performed in axial, coronal, and sagittal planes. Conventional DWI with b-values of 0 and 1000 s/mm^2^ was also performed in the axial plane. The ultra-high b-value DWI was performed with b-values of 3000 s/mm^2^ in the axial plane. Both axial T2WI and DWI were obtained at the same slice location. Dynamic contrast-enhanced (DCE) MRI was also performed. Fifteen ml of Gadobenate dimeglumine (MultiHance; Bracco Sine, Shanghai, China) was injected intravenously at a rate of 3 ml/s using a power injector (Spectris; MedRad, Warrendale, PA), followed by a 20 ml saline flush. DCE-MRI was performed in the transverse plane at baseline (pre-contrast) and during arterial, venous, and delayed phases. MR imaging parameters are shown in Table 1.

**Table 1:** MR imaging acquisition parameters

Protocol	Sequence	TR(msec)	TE(msec)	FA	FOV(cm)	Matrix	Section thickness	Intersection gap	NEX	Bandwidth
Axial T2WI	FR FSE	6627	115.1	90	20 × 20	288 × 288	3 cm	0.5 cm	2.0	62.5kHZ
Coronal T2WI	FR FSE	7229.6	115.1	90	18 × 18	288 × 288	3 cm	0.5 cm	2.0	62.5kHZ
Sagittal T2WI	FR FSE	12,333.1	108.9	90	22 × 22	288 × 288	3 cm	0.5 cm	1.5	83.31kHZ
DWI(b = 1000 s/mm^2^)	SE-EPI	2700	76.8	90	36 × 32	130 × 128	3 cm	0.5 cm	6	250kHZ
DWI(b = 3000 s/mm^2^)	SE-EPI	2700	76.8	90	32 × 32	130 × 128	3 cm	0.5 cm	6	250kHZ
Axial LAVA-Flex	3D–LAVA	3.9	1.8	15	40 × 36	288 × 192	4 cm	−2.0 cm	0.71	166.7kHZ
Contrast-enhanced imaging	3D–LAVA	2.7–4.4	1.3–2.0	15	38–40 × 30–40	288 × 256	4 cm	−0.5-0 cm	0.69–0.73	125–142.9kHZ

*FR FSE* fast recovery fast spin echo, *SE/EPI* spin-echo echo-planar imaging, *3D/LAVA* three-dimensional liver acquisition with volume acceleration, *TE* echo time, *TR* repetition time, *FA* flip angle, *FOV* field of view

**Fig. 1:**
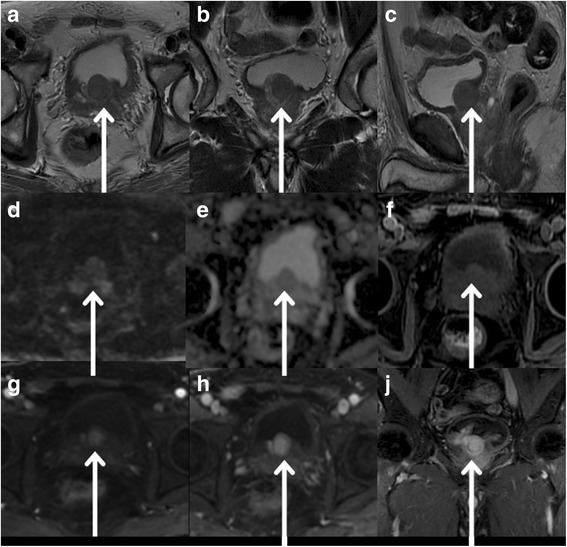
MR images of prostatic leiomyoma. **a**, **b**, **c** Axial, Coronal, and Sagittal High-resolution T2WI images show a well-circumscribed nodule with a capsule (homogeneous low signal intensity) in the bottom of prostatic left inner zone as homogeneous and slightly hypointense signal compared to the muscle (arrows). **d** On ultra-high b-value DWI (b = 3000 s/mm^2^), slightly high signal nodule compared to the muscle (arrows) was observed. **e** On ultra-high b-value DWI apparent diffusion coefficient map, ADC value was 0.817±0.016 × 10^−3^ mm^2^/s. **f** On T1WI MRI with fat suppression (Pre scanned imaging), the homogeneous isointense signal of nodule compared to the muscle was found. **g**, **h**, **j** On Axial contrast T1WI during the arterial phase, the tumor was strongly and homogeneously enhanced. During venous and delay phases, the tumor showed continuous signal increase (arrows). The margin of tumor was clear, and the envelope was not enhanced

Conventional MRI displayed a round tumor in the prostatic bottom protruding into the bladder with an isointense signal compared to the muscle on T1-weighted images (T1WI) with fat suppression and a homogeneous lesion with slightly hypointense signal on T2WI (Fig. 1). Ultra-high b-value DWI (b = 3000 s/mm^2^) features showed slightly homogeneous hyperintense signal compared to the muscle, and the mean ADC value was 0.817 ± 0.016  × 10^−3^ mm^2^/s. The tumor was strongly and homogeneously enhanced during the arterial phase with continuous signal increase of mass in the venous and delayed phases (a persistent pattern). Normal prostatic glandular tissue with a non-enhanced capsule around the mass was clearly distinguished from the tumor as a homogeneous isointense signal compared to the muscle. No central necrosis was demonstrated in the tumor.

**Fig. 2:**
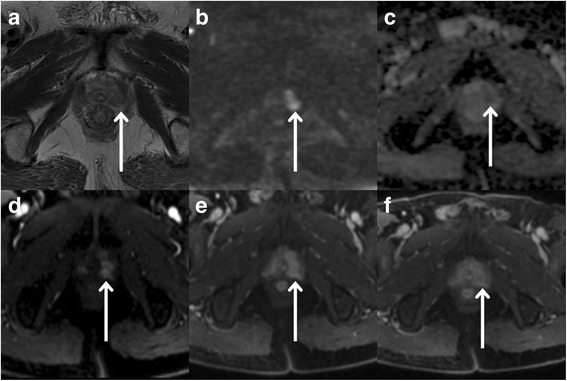
MR images of prostate cancer. **a** High-resolution T2WI shows low signal intensity area in the left transition (TZ) and peripheral zone (PZ) (arrows). **b** On ultra-high b-value DWI (b = 3000 s/mm^2^), left-side lesions were observed as significantly higher signal nodules (arrows). **c** On ultra-high b-value DWI (b = 3000 s/mm^2^) ADC map, ADC value of the left-side lesion was 0.517 ± 0.015 × 10^−3^ mm^2^/s (arrows). **d** On axial contrast T1WI during the arterial phase, the tumor was enhanced strongly and non-homogeneously (arrows). **e, f** On axial contrast T1WI during venous and delay phases, the tumor was enhanced strongly and non-homogeneously (continuous signal increase, arrows). The margin of tumor was not clear, and there was no envelope

In addition, a low signal intensity area on T2WI showed significantly higher signal nodule on ultrahigh b-value DWI, and the mean ADC value was 0.517 ± 0.015 × 10^−3^ mm^2^/s. It was found in the left transition and peripheral zone. The tumor showed clear enhancement with an irregular boundary and without capsule on DCE (Fig. 2). At the same time, prostate benign hyperplasia nodules were also found in the middle area of right transition zone, and these features were similar to the prostatic leiomyoma observed on T2WI, T1WI and DWI. The mean ADC value was 0.710 ± 0.049 × 10^−3^ s/mm^2^. The nodular was enhanced heterogeneously during the arterial phase with continuous signal increase of mass in the venous and delayed phases (a persistent pattern). There was no capsule. The preoperative MRI diagnosis revealed prostatic cancer with benign prostatic hyperplasia; however, the prostatic leiomyoma was not considered at the initial diagnosis.

Ultrasound guided systematic transperineal prostatic biopsy and subsequent histological analysis of biopsy samples confirmed prostatic cancer in the left zone (Gleason score 3 + 3 = 6).

A robot-assisted laparoscopic radical prostatectomy was performed to remove the whole prostate. In addition, histopathological examinations revealed leiomyoma based on following features: firstly, the tumor was composed primarily of hyalinized connective tissue containing spindle cells; secondly, spindle cells were arranged with a fascicular and whorled manner in foci. The cancer was found in peripheral and transitional zones of the left posterior lobe.

## Discussion and conclusions

Most cases of prostatic leiomyoma are diagnosed as incidental findings related to the history of progressive prostatism. The first case report of a prostatic leiomyoma found at autopsy was published in 1876. On that report, prostatic leiomyoma was defined as a circumscribed or encapsulated mass of smooth muscle with 1 cm or more in diameter containing varying amounts of fibrous tissue but devoid of glandular elements, which was either obviously prostatic or juxtaprostatic in origin and position [5, 6]. However, the pathogenesis of such tumor remains unclear. Several theories have described that it is formed as a result of chronic inflammatory reactions or embryological ability [6, 7] and may originate from smooth muscle elements of the periglandular prostatic tissue, prostatic capsule, or Mullerian duct remnant. The main clinical symptom is urinary disturbance, as reported in case of benign prostatic hypertrophy differing in localization within the prostate. Occasionally, it may present as a large mass bulging into the rectum producing rectal symptoms [5, 8]. This disease is benign and surgical resection is recommended if present with severe clinical symptoms.

Typical imaging features of prostatic leiomyoma on contrast-enhanced CT have been reported in some cases, which show a round hypodense mass and homogeneous enlargement of the prostate with well circumscribed appearance [8–11]. Only a few studies have reported that typical MRI features of prostatic leiomyoma show an isointense and homogeneous signal mass on T1WI, slightly hyperintense or hypointense on T2WI, and homogeneous and strongly enhanced lesion on contrast-enhanced MR images [11–13], The manifestation of our case was compatible with those criteria reported in the literature. In contrast, Ringoir and colleagues have reported a heterogeneous prostatic leiomyoma with cystic and solid components in the right prostate lobe on MRI [14]. To the best of our knowledge, ultra-high b-value DWI and ADC features of the prostatic leiomyoma have not been reported in the literature till date. Only Mussi et al. have reported 3 cases with low and intermediate b-values DWI and ADC maps (b values = 50, 400 and 800) [15]. In our case report, typical features of the prostatic leiomyoma were slightly homogeneous hyperintense signal compared to the muscle on DWI and a higher ADC value as compared to the prostate cancer, which altogether reflecting pathological characteristics of abundant muscle or fibrous tissue component.

The major differential diagnosis of prostatic leiomyoma is prostate cancer. DWI is a functional technique enabling qualitative and quantitative assessment of the prostate cancer with high diagnostic accuracy. Prostatic cancer shows a higher signal intensity on DWI and a lower ADC value as compared to normal prostatic tissue [16, 17]. The American College of Radiology and European Society of Urogenital Radiology guidelines have recommended the use of a high b-value (≥ 1400 s/mm^2^) as it suppresses the signal of normal prostatic tissue effectively; thus, the contrast between cancerous and non-cancerous lesions can be emphasized better on DWI. It is difficult to differentiate prostatic leiomyoma from prostate cancer while using low and intermediate b-values DWI and ADC maps (b values = 50, 400 and 800). If the MR scanner yields an adequate signal-to-noise ratio, the use of a very high b-value (e.g. 2000 s/mm^2^) is more advantageous for cancer detection [18, 19]. Zhang, et al. have reported that DWI with an ultra-high b-value of 3000 s/mm^2^ is found to be the most accurate and reliable MRI modality for prostatic cancer detection and localization [20]. ADC values can be useful for the characterization of clinically significant cancer. The similar signal feature was seen in the prostatic leiomyoma and prostate cancer on T2WI in this case, but the ADC value of prostate cancer was lower than that of leiomyoma. In addition, DCE-MRI added only little information for differentiating between prostate leiomyoma and prostate cancer. Multiparametric-MRI can detect clinically significant prostate cancer with high accuracy [21]. In this case, the differentiation of leiomyoma from prostatic cancer may be easily done by MRI findings alone.

However, MRI features of stromal hyperplastic nodules of benign prostatic hyperplasia (BPH) are similar to the prostatic leiomyoma; thus, it is poorly identified because of the overlapping of conventional imaging features [22]. Patch and Rhea have pointed out that most cases with combined leiomyoma and BPH can be missed on MRI, and 25.4% of 181 consecutive patients with total prostectomy show leiomyoma in benign hyperplastic nodules [23]. Stromal hyperplastic nodules and prostatic leiomyoma are benign and non-cancerous lesions.

The final diagnosis also depends on pathological examinations. The most commonly evidenced diagnostic method is B-ultrasound guided transrectal prostate biopsy or transurethral resection of the prostate. Macroscopically, a leiomyoma relates to a well-defined nodular formation [24]. Microscopically, it is characterized by intersecting bundles not atypical, smooth muscle cells with uniform cigar-shaped, and slightly vesicular nuclei. The spindle cell population is desmin, smooth muscle actin (SMA), and androgen receptor positive [12]. To date, no reports have indicated recurrence of prostatic leiomyomas after surgical removal [8].

In conclusion, we report a relatively rare leiomyoma of the prostate gland. Upon observation of a well-circumscribed tumor with no enhanced capsule with homogeneous and almost isointense signal compared to the muscle on T1WI and slightly hypointense signal on T2WI with a strong and homogeneous enhancement by DCE-MRI in the prostatic inner zone, leiomyoma should be considered in the differential diagnosis.

## Abbreviations

ADC: Apparent diffusion coefficient
BPH: Benign prostatic hyperplasia
DCE: Dynamic contrast enhancement
DWI: Diffusion weighted imaging
MRI: Magnetic resonance imaging
SH: Stromal hyperplasia
SMA: Smooth muscle actin
T1WI: T1-weighted image
T2WI: T2-weighted image
